# *Dendrobium officinale* alleviates high-fat diet-induced nonalcoholic steatohepatitis by modulating gut microbiota

**DOI:** 10.3389/fcimb.2023.1078447

**Published:** 2023-02-13

**Authors:** Gege Tian, Wei Wang, Enrui Xia, Wenhui Chen, Shunzhen Zhang

**Affiliations:** ^1^ College of Chinese Materia Medica, Yunnan University of Chinese Medicine, Kunming, China; ^2^ The Key Laboratory of Microcosmic Syndrome Differentiation, Education Department of Yunnan, Yunnan University of Chinese Medicine, Kunming, China; ^3^ College of Basic Medicine, Yunnan University of Chinese Medicine, Kunming, China

**Keywords:** *dendrobium officinale*, nonalcoholic steatohepatitis, gut microbiota, lipopolysaccharide, intestinal permeability, liver inflammation, traditional chinese medicine

## Abstract

**Introduction:**

The gut microbiota plays an important role in the development of nonalcoholic steatohepatitis (NASH). This study investigated the preventive effect of *Dendrobium officinale* (DO), including whether its effect was related to the gut microbiota, intestinal permeability and liver inflammation.

**Methods:**

A NASH model was established in rats using a high-fat diet (HFD) and gavage with different doses of DO or Atorvastatin Calcium (AT) for 10 weeks. Body weight and body mass index along with liver appearance, weight, index, pathology, and biochemistry were measured to assess the preventive effects of DO on NASH rats. Changes in the gut microbiota were analyzed by 16S rRNA sequencing, and intestinal permeability and liver inflammation were determined to explore the mechanism by which DO treatment prevented NASH.

**Results:**

Pathological and biochemical indexes showed that DO was able to protect rats against HFD-induced hepatic steatosis and inflammation. Results of 16S rRNA sequencing showed that Proteobacteria, *Romboutsia, Turicibacter, Lachnoclostridium, Blautia, Ruminococcus_torques_group, Sutterella, Escherichia-Shigella, Prevotella, Alistipes*, and *Lactobacillus_acidophilus* differed significantly at the phylum, genus, and species levels. DO treatment modulated the diversity, richness, and evenness of gut microbiota, downregulated the abundance of the Gram-negative bacteria Proteobacteria, *Sutterella*, and *Escherichia-Shigella*, and reduced gut-derived lipopolysaccharide (LPS) levels. DO also restored expression of the tight junction proteins, zona occludens-1 (ZO-1), claudin-1, and occludin in the intestine and ameliorated the increased intestinal permeability caused by HFD, gut microbiota such as *Turicibacter*, *Ruminococcus*, *Escherichia-Shigella*, and *Sutterella*, and LPS. Lower intestinal permeability reduced LPS delivery to the liver, thus inhibiting TLR4 expression and nuclear factor-kappaB (NF-κB) nuclear translocation, improving liver inflammation.

**Discussion:**

These results suggest that DO may alleviate NASH by regulating the gut microbiota, intestinal permeability, and liver inflammation.

## 1 Introduction

Nonalcoholic steatohepatitis (NASH) is the inflammatory subtype of nonalcoholic fatty liver disease (NAFLD), defined by the simultaneous appearance of more than 5% fat accumulation, hepatocyte injury (ballooning), and inflammation, with or without fibrosis (Kanwal et al., 2021). The prevalence of NASH is increasing and is predicted to rise by 63% by 2030 (Estes et al., 2018). More than 20% of NASH patients develop irreversible cirrhosis or hepatocellular carcinoma (HCC) (Sheka et al., 2020). The “multiple hit” hypothesis is the currently accepted explanation of the complex etiology and pathophysiology of NAFLD (Salvoza et al., 2022). The “multiple hit” pathogenesis of NASH is closely related to the composition of the gut microbiota and intestinal permeability which can influence the development of NASH by regulating liver inflammation(Zhu et al., 2021). Effectively controlling NASH is critical to prevent the development of cirrhosis or HCC. While NAFLD-specific drug research is primarily focused on NASH, however, no specific drugs have been approved by the Food and Drug Administration or European Medicines Agency (Fraile et al., 2021). Lifestyle changes such as healthy eating and physical exercise are suggestions for treating NASH, however, these methods are not always reliable.

Potent natural products such as Traditional Chinese Medicine (TCM), a conventional and effective therapeutic strategy associated with few side effects, are shown to improve gut microbiota and inhibit NASH progression (Chen M, et al., 2021). *Dendrobium officinale* (DO), a plant that is widely used as a TCM and homologous food product, contains many compounds, including polysaccharides, phenanthrenes, and bibenzyls (Chen WH, et al., 2021), with a variety of pharmacological effects such as lowering lipid content, regulating gut microbiota, protecting the liver, preventing inflammation and hypoglycemia, and protecting intestinal health (Wang K, et al., 2020; Yang J, et al., 2020; Liu et al., 2021; Fang et al., 2022). DO can also alleviate lipopolysaccharides (LPS)-induced gastric cancer cell injury by inhibiting TLR4 signaling and can reverse intestinal epithelial cell damage by regulating TLR4 (Zhang et al., 2019; Yang K, et al., 2020).

The impact of DO on NASH remains unknown. Polysaccharides are the pharmacologically active ingredient of DO and while not easily digested and absorbed, it is able to regulate gut microbiota (Li et al., 2019). Our previous network pharmacological studies also identified TLR4 as a possible target for DO in the treatment of NASH (**Supplementary Material**). Gut-derived LPS, intestinal permeability, and the receptor TLR4 of LPS are the critical mechanisms by which gut microbiota impact the development of NASH (Xiang et al., 2022). Patients with NASH often have an imbalanced gut microbiota, increased intestinal permeability, high LPS levels, and elevated expression of liver TLR4 (Abdel-Razik et al., 2018; Ghetti et al., 2019; Craven et al., 2020). The gut and liver have bidirectional communication across the portal vein and alterations in the balance of microbial populations and function, known as dysbiosis, can disrupt the intestinal barrier tight junctions (Albillos et al., 2020; Bauer et al., 2022). This morphological alteration leads to increased intestinal permeability and allows the translocation of bacteria and/or bacterial products such as LPS through the portal vein to the liver (Plaza-Díaz et al., 2020). The gut microbiota is a source of Toll-like receptor (TLR) ligands, and compositional changes in the microbiota can increase the delivery of TLR ligands to the liver (Miura and Ohnishi, 2014). TLR4 is widely distributed in liver cells, is involved in several liver diseases, and plays a key role in inflammatory pathogenesis following activation by bacteria and/or bacterial products (Wang Y, et al., 2020). TLR4 is a natural receptor of LPS and LPS-induced activation of TLR4 leads to NF-KB nuclear translocation, promotes the release of proinflammatory factors such as IL-6 and TNF-α, and induces the progression from simple fatty liver disease to NASH (Heida et al., 2021). Indeed, in TLR4 knockout NASH mice, liver inflammation and fibrosis are significantly reduced (Csak et al., 2011). Thus, the current study sought to assess whether DO can regulate gut microbiota, intestinal permeability, and liver inflammation to alleviate NASH.

## 2 Materials and methods

### 2.1 Chemicals, reagents, and materials

DO powder was purchased from Yunnan Tianbao Betula Biological Resources Development Co., Ltd. (Yunnan, China) and Atorvastatin calcium (AT) tablets were purchased from Beijing Jialin Pharmaceutical Co., Ltd. (Beijing, China). The normal diet was purchased from Jiangsu Medisen Biological Medicine Co., Ltd. (Bei Jing, China). Kits used to measure alanine transaminase (ALT), aspartate transaminase (AST), triglyceride (TG), total cholesterol (TC), low-density lipoprotein cholesterol (LDL-c), high-density lipoprotein cholesterol (HDL-c), and gamma-glutamyl transpeptidase (GGT) and the total protein assay were purchased from the Nanjing Jiancheng Bioengineering Institute (Nanjing, China). Kits used to measure interleukin-6 (IL-6), interleukin 1-β (IL-1β), LPS, tumor necrosis factor-α (TNF-α), and diamine oxidase (DAO) were purchased from the Jiangsu Meimian Industrial Co., Ltd. (Jiangsu, China). D-lactate (D-LA) kit was purchased from Jiangsu Addison Biotechnology Co., Ltd. (Jiangsu, China). Anti-Occludin rabbit pAb, anti-Claudin-1 rabbit pAb, anti- Zona occludens-1 (ZO-1) rabbit pAb, and anti-NF-kB p65 (p 65) rabbit pAb were purchased from Servicebio (Wuhan, China). Ultrapure RNA Kit, cDNA Synthesis Kit, and UltraSYBR Mixture were purchased from CoWin Biosciences (Jiangsu, China). TLR4 rabbit pAb was purchased from Proteintech Group, Inc (Rosemont, USA). Phospho-NF-kB p65 (p-p65) antibody was purchased from Affinity Biosciences Ltd. (OH, USA). Anti-beta actin (β-actin) antibody was obtained from Abcam Inc. (Cambridge, UK).

### 2.2 Animals and experimental design

All experimental procedures followed the guidelines of the Animal Ethics Committee of the Yunnan University of Chinese Medicine (Approval lot: R-062021024).

Healthy male Sprague-Dawley (SD) rats (180–200 g; SPF) were provided by Hunan Slike Jingda Laboratory Animal Co., Ltd. (Hunan, China). The rats were maintained in a specific pathogen-free standard environment on the public platform for animal experiments in the Science and Technology Department of the Yunnan University of Chinese Medicine. The rearing temperature was 20–25°C and the relative humidity was 50% ± 10%, with 12 hours of alternating light. All experimental rats had free access to distilled water and were fed a normal or a high-fat diet (HFD, 82.5% normal diet, 10% lard, 2% cholesterol, 0.5% sodium cholate, and 5% egg yolk powder).

After 1 week of adaptive feeding, the rats were randomized into the following six groups (n = 8 rats per group): 1) Control group, fed with a normal diet and gavaged with distilled water; 2) HFD group, fed with a HFD and gavaged with distilled water; 3) AT group, fed with a HFD and gavaged with 20 mg/(kg·d) AT; 4) High-dose DO group (DOH), fed with a HFD and gavaged with 1000 mg/(kg·d) DO powder; 5) Middle-dose DO group (DOM), fed with a HFD and gavaged with 500 mg/(kg·d) DO powder; 6) Low-dose DO group (DOL), fed with a HFD and gavaged with 250 mg/(kg·d) DO powder. AT and DO powder were prepared separately using distilled water. Rats were gavaged with the corresponding drug (or distilled water) once a day for 10 weeks.

### 2.3 Sample collection

Body weights were recorded weekly during the experiment. After the last administration at the end of 10 weeks, rat feces were collected from the anus of each rat using sterile EP tubes and immediately preserved in liquid nitrogen. Rats were euthanized after fasting for 12 hours, and liver tissue, small intestine tissue, and serum samples were collected. Serum samples and a portion of both the liver and small intestine tissues were stored at -80°C.

### 2.4 Serum and hepatic biochemical assay

Serum AST, ALT, GGT, TG, TC, LDL-c, HDL-c, and D-LA levels and liver TG, TC, LDL-c, and HDL-c levels were measured using a commercial kit.

### 2.5 Enzyme-linked immunosorbent assay

LPS levels in the liver, serum, and ileum, IL-6, IL-1β, TNF-α levels in the liver and ileum, and DAO levels in the ileum were detected using ELISA kits.

### 2.6 Histopathological analysis

Liver and ileum tissues fixed in 4% paraformaldehyde solution were dehydrated with different concentrations of ethanol, made transparent with xylene, embedded in liquid paraffin, stained with H&E, and sealed with neutral gum. The fixed liver tissue was dehydrated in different concentrations of sucrose solution, embedded in an optimal cutting temperature compound, sliced using a cryostat, stained with oil red O, and sealed with glycerol gelatin. A slide scanning image analysis system (Shenzhen Shengqiang Technology, China) was used to observe the staining of the pathological sections at 400x, and the oil red O-positive area was analyzed by ImageJ software (NIH, Bethesda, MA, USA).

### 2.7 Western blot

TLR4, p-p65 and p65 expression in liver tissues were determined by Western blot. Liver tissue (50 mg) and 0.5 ml RIPA lysate were added to an EP tube, ground for 60 s, and centrifuged at 4°C at 10,000×g for 10 min. BCA protein quantification was used to measure the protein concentration. The protein solution was added to a 5x reduced protein loading buffer at a ratio of 4:1 and denatured in a boiling water bath for 15 min. Electrophoresis was conducted at 80V for 20 min and then at 120V until the bromophenol blue ran to a position 1 cm from the lower end of the glass plate. TLR4 (1:8000), p-p65 (1:1000), p65 (1:1000), and β-Actin (1:2000) were incubated for 60 min and washed with TBST until no skimmed milk powder was present. The universal secondary antibodies (1:5000) were incubated for 60 min at room temperature and washed three times with TBST for 5 min each. Immunoreactive protein bands were visualized with a chemiluminescence HRP substrate using a ChemiDoc XRS image detector (Jena Analytical Instruments AG, Jena, Germany). The blots were analyzed using ImageJ software.

### 2.8 Immunohistochemistry

Sections of paraffin-embedded ileum tissue were deparaffinized and rehydrated. Antigen was repaired using citric acid antigen repair buffer and 3% hydrogen peroxide was used to block any endogenous peroxidase. BSA was added dropwise for serum blocking followed by the addition of ZO-1 (1:1000), occludin (1:1000), or claudin-1 (1:800). After incubating the samples overnight, a secondary antibody was added dropwise. The colour was developed with DBA, the cell nuclei were re-stained, and the samples were dehydrated to seal the slides. A slide scanning image analysis system (Shenzhen Shengqiang Technology, China) was used at 400x to observe the samples. Ultimately, the Image-Pro Plus software (U.S. MEDIA CYBERNETICS) was used to count the mean density and analyze the integrated optical density (IOD) of positive staining.

### 2.9 16S rRNA gene sequencing and analysis

#### 2.9.1 DNA extraction and PCR amplification

Total microbial genomic DNA was extracted from rat feces samples using the E.Z.N.A.^®^ Stool DNA Kit (Omega Bio-tek, Norcross, GA, U.S.). The quality and concentration of DNA were determined by 1.0% agarose gel electrophoresis and a NanoDrop^®^ ND-2000 spectrophotometer (Thermo Scientific Inc., USA) and kept at -80 °C prior to further use. The hypervariable region V3-V4 of the bacterial 16S rRNA gene were amplified with primer pairs 338F (5’-ACTCCTACGGGAGGCAGCAG-3’) and 806R(5’-GGACTACHVGGGTWTCTAAT-3’) by an ABI GeneAmp^®^ 9700 PCR thermocycler (ABI, CA, USA). The PCR reaction mixture including 4 μL 5 × Fast Pfu buffer, 2 μL 2.5 mM dNTPs, 0.8 μL each primer (5 μM), 0.4 μL Fast Pfu polymerase, 10 ng of template DNA, and ddH2O to a final volume of 20 µL. PCR amplification cycling conditions were as follows: initial denaturation at 95 °C for 3 min, followed by 27 cycles of denaturing at 95 °C for 30 s, annealing at 55 °C for 30 s and extension at 72 °Cfor 45 s, and single extension at 72 °C for 10 min, and end at 4 °C. All samples were amplified in triplicate. The PCR product was extracted from 2% agarose gel and purified using the AxyPrep DNA Gel Extraction Kit (Axygen Biosciences, Union City, CA, USA) according to manufacturer’s instructions and quantified using Quantus™ Fluorometer (Promega, USA).

#### 2.9.2 Illumina MiSeq sequencing

Purified amplicons were pooled in equimolar amounts and paired-end sequenced on an Illumina MiSeq PE300 platform platform (Illumina, San Diego,USA) according to the standard protocols by Majorbio Bio-Pharm Technology Co. Ltd. (Shanghai, China). The raw sequencing reads were deposited into the NCBI Sequence Read Archive (SRA) database (Accession Number: PRJNA872008).

#### 2.9.3 Statistical analysis

Bioinformatic analysis of the gut microbiota was carried out using the Majorbio Cloud platform (https://cloud.majorbio.com). Based on the OTUs information, Species accumulation curve, rank-abundance, alpha diversity indices including observed OTUs, Chao, Qstat and Smithwilson index were calculated with Mothur v1.30.1.The similarity among the microbial communities in different samples was determined by Principal Component Analysis (PCA) based on Bray-curtis dissimilarity using Vegan v2.5-3 package. The PERMANOVA test was used to assess the percentage of variation explained by the treatment along with its statistical significance using Vegan v2.5-3 package. The linear discriminant analysis (LDA) effect size (LEfSe) (http://huttenhower.sph.harvard.edu/LEfSe) was performed to identify the significantly abundant taxa of bacteria among the different groups (LDA score > 3, *p* < 0.05).

### 2.10 Quantitative real-time-PCR analysis

Total RNA was extracted from liver tissue using Ultrapure RNA Kit and was reverse transcribed into cDNA using cDNA Synthesis Kit. The PCR cycle system was set as follows: at 95°C for 10 min, at 95°C for 15 s, and at 60°C for 60 s, for a total of 40 cycles. A total of 1μL cDNA template was used for PCR amplification using the following primers: TLR4, forward: 5′-CCGCTCTGGCATCATCTTCA-3′, reverse: 5′-TGGGTTTTAGGCGCAGAGTT-3′; GAPDH, forward: 5′-GCCCAGCAAGGATACTGAGA-3′, reverse: 5′-GGTATTCGAGAGAAGGGAGGGC′.

### 2.11 Statistical analysis

Statistical analysis was conducted using SPSS software (Version 25, SPSS Inc., Chicago, USA) and graphs were created using GraphPad Prism software (Version 9.4.0, GraphPad Software Inc., CA, USA). Data are shown as the mean ± SD. Normally distributed data were tested by one-way ANOVA followed by the LSD or Dunnett’s test. Other types of data were tested using the non-parametric Mann-Whitney test (Kruskal-Wallis test for multiple groups). Differences were considered statistically significant at a *p*-value <0.05.

## 3 Results

### 3.1 Effect of DO on the body weight and liver weight of rats

**Figure 1:**
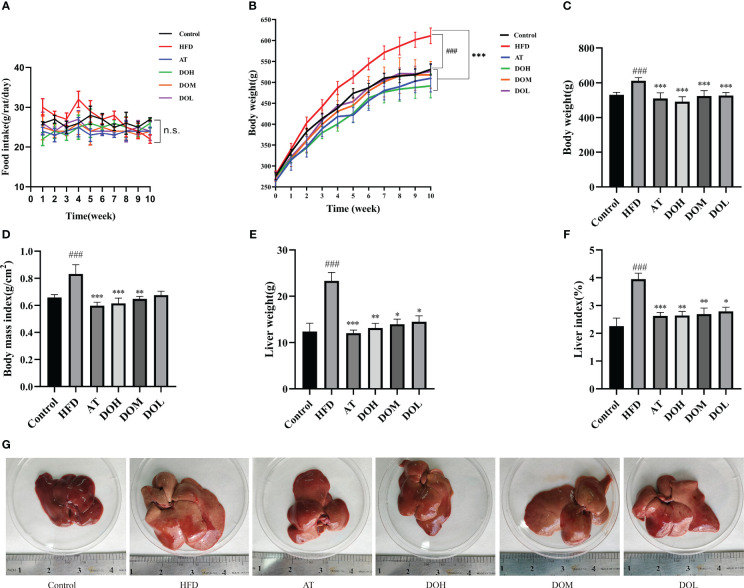
**(A)** Temporal changes in food intake by group; **(B)** temporal changes in body weight by group; **(C)** final body weight by group; **(D)** body mass index by group; **(E)** liver weight by group; **(F)** liver index by group; **(G)** representative photo of a liver from each group. All data are shown as the mean ± SD (n=8). ^#^ *p <* 0.05, ^###^ *p <* 0.001 vs. the Control group; *p < 0.05, **p < 0.01, ***p < 0.001 vs. the HFD group.

HFD rats had a higher food intake than those in the other groups at 4 weeks. While food intake gradually decreased in the last 6 weeks, possibly due to an anorexic reaction caused by overeating the HFD, there was no significant difference in intake among the groups (**Figure 1A**). The HFD rats gained weight faster than rats in the other groups (**Figure 1B**). HFD rats had significantly higher body weight (*p*-value < 0.001), body mass index (*p*-value <0.05), liver weight (*p*-value <0.001), and liver index (*p*-value <0.001) than Control rats. The AT and DO interventions significantly reduced the body weight, body mass index, liver weight, and liver index (*p*-value <0.05, <0.01, and <0.001, respectively) (**Figures 1C–F**). In addition, the livers of HFD rats were significantly larger and more yellow than those of the Control, AT, and DO rats, suggesting that HFD rat livers may have accumulated more lipids (**Figure 1G**). These results indicate that DO effectively inhibited HFD-induced weight gain, lipid deposition, and liver enlargement.

### 3.2 Effect of DO on liver pathology and biochemical parameters

**Figure 2:**
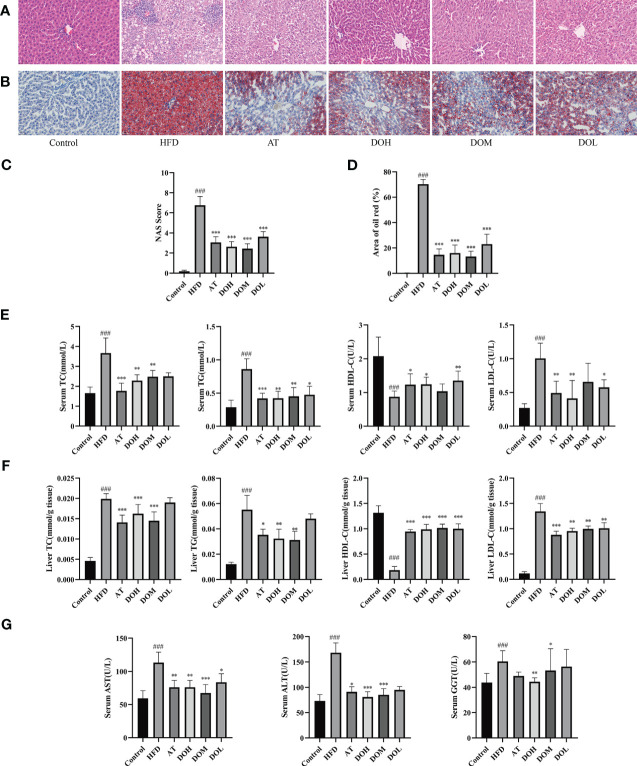
Detection of liver pathology and biochemical criteria. **(A)** Representative H&E stained liver samples by group (400x magnification); **(B)** representative images of oil red O stained liver samples by group (400x magnification); **(C)** liver NAS scores by group; **(D)** oil red O staining area by group; **(E)** serum lipid content by group; **(F)** liver lipid content by group; **(G)** serum AST, ALT and GGT levels by group. All data are shown as the mean ± SD (n =8). ^###^ *p <* 0.001 vs. the Control group; *p < 0.05, ** *p <* 0.01, ***p < 0.001 vs. the HFD group.

After 10 weeks of HFD feeding with or without DO treatment, liver cells from Control rat livers were neatly arranged, the liver cords were clear, and no obvious lipid deposition was observed. In contrast, liver cells from HFD rat livers were disordered, with more extensive and robust steatosis accompanied by intralobular inflammatory foci and balloon-like changes. Inflammation and steatosis were lower in the livers of rats in the DO and AT groups than those in the HFD group (**Figure 2A**). The NAFLD activity score (NAS), semi-quantitative data used to assess NAFLD progression (NAS >4), showed that NASH was occurring in the HFD group, indicating that a HFD successfully induced NASH. NAS scores were significantly lower following DO and AT treatment (*p*-value <0.001) (**Figure 2C**). Liver oil red O staining showed no obvious lipid deposition in Control rats and a large amount of deposition in HFD rats (*p*-value <0.001). The area of lipid deposition was decreased following DO and AT treatment (*p*-value <0.01 and <0.001, respectively) (**Figures 2B, D**). These results supported the success of NASH modeling in this study and indicated that DO treatment reduces lipid accumulation and inflammation in rat livers.

TC, TG, HDL-c, and LDL-c are clinical indicators used to reflect blood lipid levels and lipid metabolism. HFD significantly increased serum and liver TC, TG, and LDL-c and reduced HDL-c levels (*p*-value <0.001). These blood and liver lipid indexes were significantly improved following DO and AT treatment (*p*-value <0.05, <0.01, and <0.001, respectively) (**Figures 2E, F**). ALT, AST, and GGT are the most used clinical indicators of liver function. When the liver is damaged, hepatocytes produce these proteins, resulting in an increase in serum ALT, AST, and GGT levels and indicating the occurrence of liver disease and inflammation. HFD significantly increased serum ALT, AST, and GGT levels (*p*-value <0.001) and these indexes were significantly decreased following DO and AT treatment (*p*-value <0.05, <0.01, and <0.001, respectively) (**Figure 2G**).

### 3.3 Effect of DO on the gut microbiota of NASH rats

**Figure 3:**
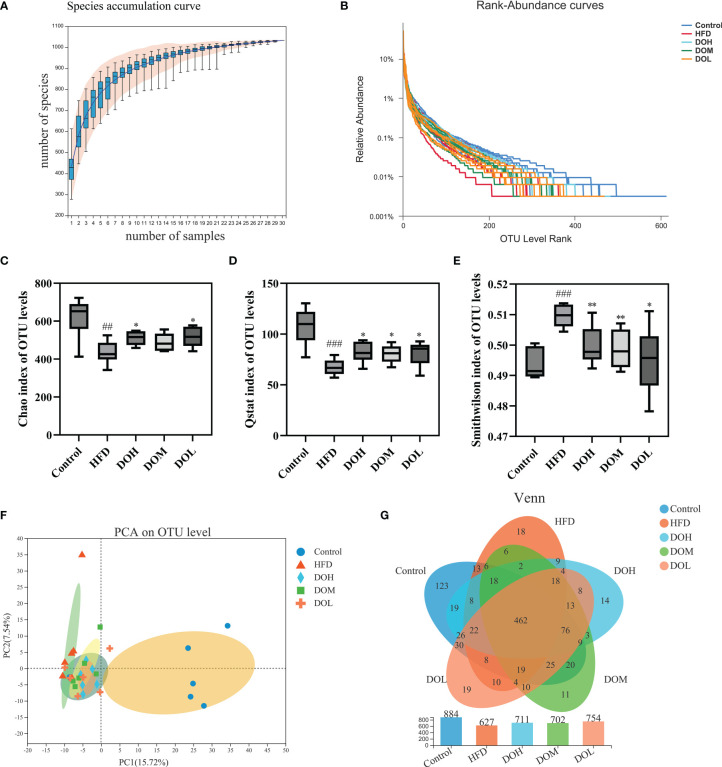
**(A)** Species accumulation curve; **(B)** rank-abundance curve; **(C)** Chao index by group; **(D)** Qstat index by group; **(E)** Smithwilson index by group; **(F)** PCA plot of microbial communities based on the OTU level. **(G)** Venn diagram based on the OTU level. Data are shown as the mean ± SD (n=6). ^##^ *p <* 0.01, ^###^ *p <* 0.001 vs. the Control group; *p < 0.05, **p < 0.01 vs. the HFD group.

Structural changes in the gut microbiota of rats that received DO treatment were assessed using 16S rRNA sequencing analysis. A total of 1,548,295 sequences were obtained from 30 samples, and 1,033 OTUs were collected at a 97% similarity level. The species accumulation and rank-abundance curves showed that most of the diversity and species were included and the amount of sequencing data was adequate (**Figures 3A, B**).

### 3.4 Effect of DO on the Alpha diversity and Beta diversity of rat gut microbiota

Alpha diversity was determined using Chao, Qstat, and Smithwilson to calculate the complexity of species diversity in samples. The Chao, Qstat, and Smithwilson indexes describe the richness, diversity, and evenness of the gut microbiota, respectively. In the current study, changes in these indexes are shown in **Figures 3C–E**. The Chao (*p*-value <0.01) and Qstat (*p*-value <0.001) were lower and the Smithwilson (*p*-value <0.001) was higher in the HFD group than in the Control group, suggesting that a HFD can significantly reduce microbiome richness, diversity, and evenness. The Chao and Qstat increased and the Smithwilson decreased following DO treatment (*p*-value <0.05 and <0.01, respectively) suggesting that DO may effectively improve the diversity, richness, and evenness of gut microbiota in NASH rats.

Beta diversity principal component analysis (PCA) was performed to clarify the effects of HFD and DO intervention on the composition and structure of the gut microbiota. As expected, PCA revealed a clear separation between the Control and HFD groups, and the composition of the gut microbiota exhibited a clear response to DO intervention (**Figure 3F**). The unique and common OTUs in the Venn diagram more directly indicated the unique species information of each group. Different numbers of OTUs were detected in each group, including 884 in the Control group, 627 in the HFD group, 711 in the DOH group, 702 in the DOM group, and 754 in the DOL group. A total of 462 OTUs were shared by all groups and each group had unique OTUs. These results showed that DO could ameliorate the gut microbiota disorder induced by HFD in NASH rats (**Figure 3G**).

### 3.5 Effect of DO on the gut microbiota at the phylum, genus, and species levels

**Figure 4:**
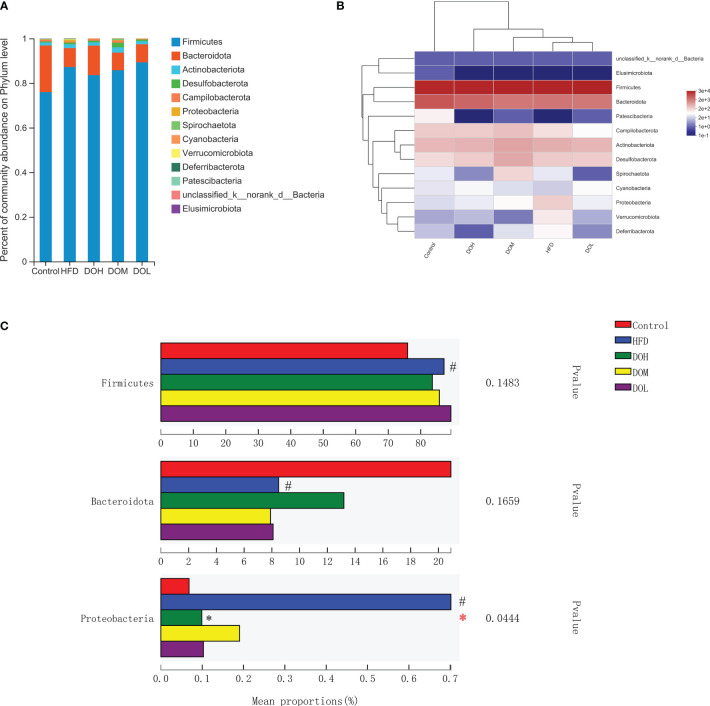
DO treatment modulated the gut microbiota composition at the phylum level. **(A)** Community abundance at the phylum level (%); **(B)** heat map of cluster stacking at the phylum level; **(C)** the relative abundances of Firmicutes, Bacteroidetes, and Proteobacteria. Data are expressed as the mean ± SD (n=6). ^#^ *p <* 0.05 vs. the Control group; *p < 0.05 vs. the HFD group.

To evaluate the effect of DO on the gut microbiota of NASH rats, the microbial abundance at the phylum, genus, and species levels was determined using taxonomic analysis. At the phylum level, 13 phyla were found in five groups, of which Firmicutes and Bacteroidetes accounted for the largest proportion. The abundance of Firmicutes, Bacteroidetes, and Proteobacteria related to NASH was changed (**Figure 4A**). Proteobacteria were positively correlated with the HFD group (**Figure 4B**). While the relative abundances of Firmicutes and Proteobacteria were higher in the HFD group than in the Control group (*p <*0.05), Bacteroidetes were lower in the HFD group (*p <*0.05). After DO intervention, the relative abundance of Proteobacteria was significantly decreased in the DOH group (*p <*0.05) (**Figure 4C**). These results indicated that compared with the Control group, the composition of gut microbiota in the HFD group changed significantly at the phylum level, especially for Proteobacteria.

**Figure 5:**
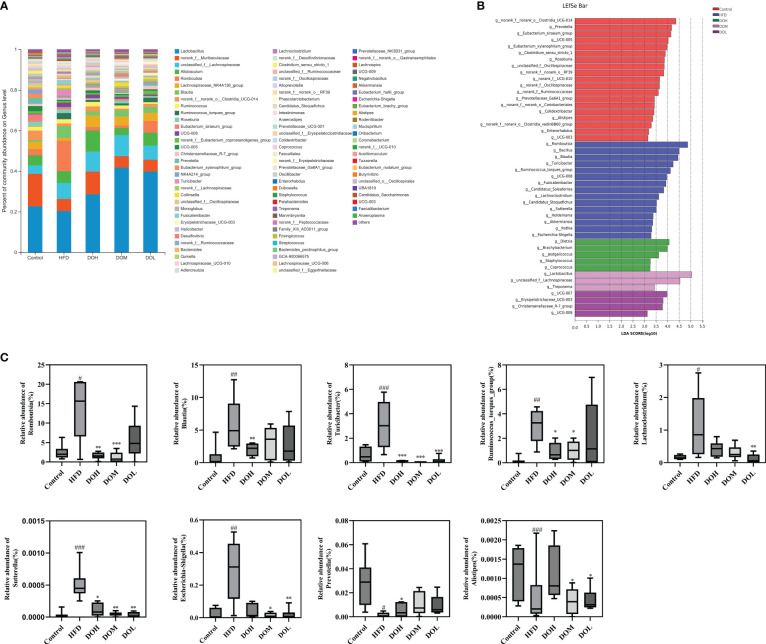
DO treatment modulated the composition of the gut microbiome at the genus level. **(A)** Community abundance at the genus level (%); **(B)** LEfSe of the gut microbiota at the genus level; **(C)** relative abundances of *Romboutsia*, *Blautia, Turicibacter, Ruminococcus_torques_group, Lachnoclostridium, Sutterella, Escherichia-Shigella, Prevotella*, and *Alistipe*. Data are expressed as the mean ± SD (n=6). ^#^ *p <* 0.05, ^##^ *p*< 0.01, ^###^ *p *< 0.001 vs. the Control group; *p < 0.05, **p < 0.01, ***p < 0.001 vs. the HFD group.

**Figure 6:**
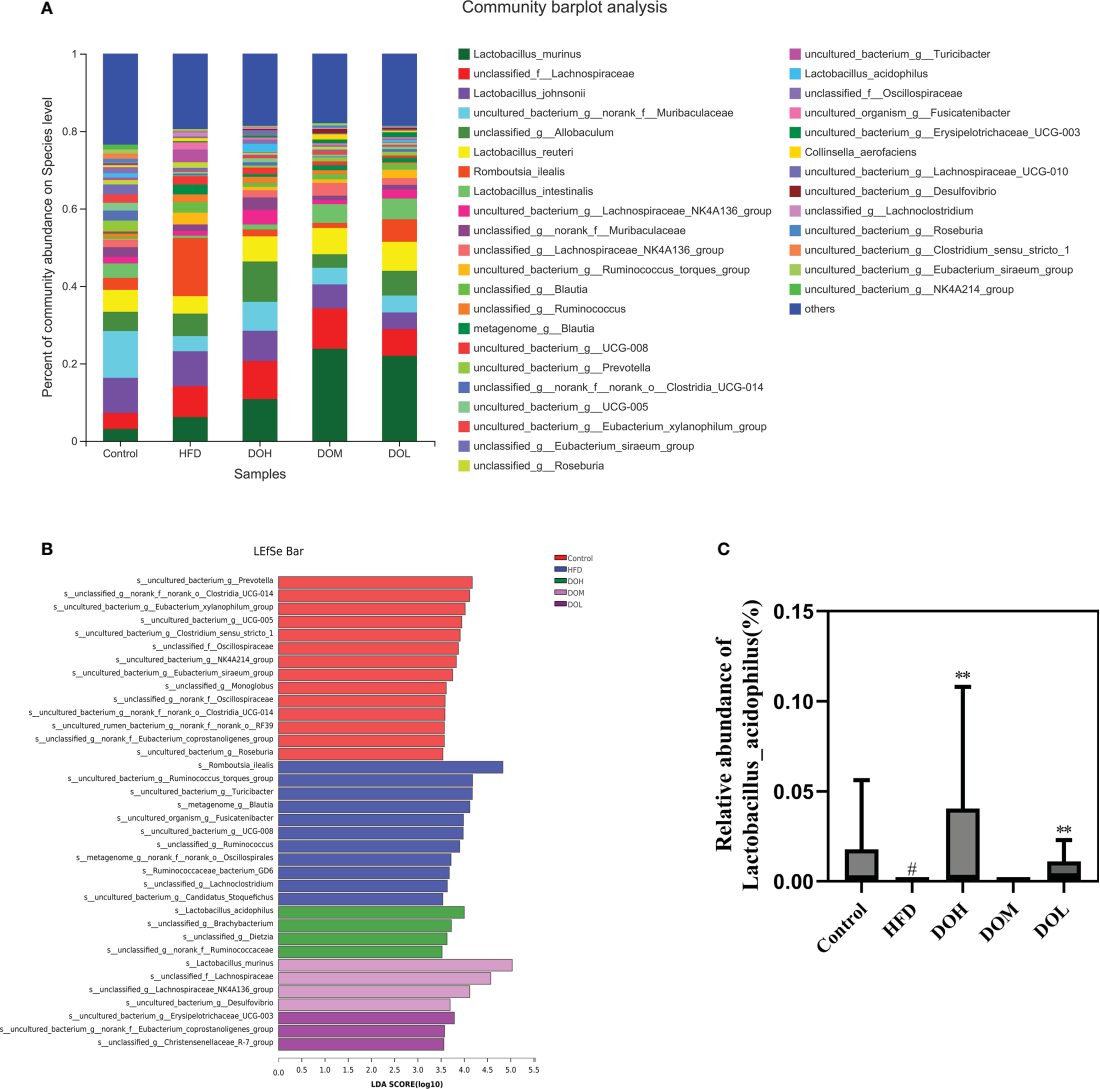
DO treatment modulated the gut microbiota composition at the species level. **(A)** Community abundance at the species level (%); **(B)** LEfSe of the gut microbiota at the species level; **(C)** relative abundances of *Lactobacillus_acidophilus*. Data are expressed as the mean ± SD (n=6). ^#^ *p <* 0.05 vs. the Control group; **p < 0.01 vs. the HFD group.

The abundance of gut microbiota at the genus and species levels also differed by group (**Figure 5A**, **6A**). To identify specific bacterial taxa that arose after DO supplementation, LEfSe analysis (all-against-all) with a 3.0 threshold for discriminative features on the logarithmic LDA scale was performed. In LEfSe, different colours represent different groups. The potentially harmful bacteria, *Romboutsia, Turicibacter, Lachnoclostridium, Blautia, Ruminococcus_torques_group, Sutterella*, and *Escherichia-Shigella* were enriched in the HFD group at the genus level (**Figure 5B**). DO treatment reduced the abundance of *Romboutsia, Turicibacter, Lachnoclostridium, Blautia, Ruminococcus_torques_group, Sutterella*, and *Escherichia-Shigella* than the HFD group (*p*-value <0.05, <0.01, and <0.001, respectively) (**Figure 5C**). Meanwhile, the abundance of the potentially beneficial bacteria, *Prevotella* and *Alistipes*, was significantly lower in the HFD group than in the Control group (*p*-value <0.05 and <0.001, respectively), and DO intervention increased the levels of these organisms (*p*-value <0.05) (**Figure 5C**). At the species level, the probiotic *Lactobacillus_acidophilus* was significantly enriched in the DOH group (**Figure 6B**). The HFD group had a significantly lower abundance of *Lactobacillus_acidophilus* than the Control group (*p*-value <0.05), and the level of this bacteria increased significantly in the DOH and DOL groups (*p*-value <0.01) (**Figure 6C**).

### 3.6 Effect of DO on intestinal permeability of rats

**Figure 7:**
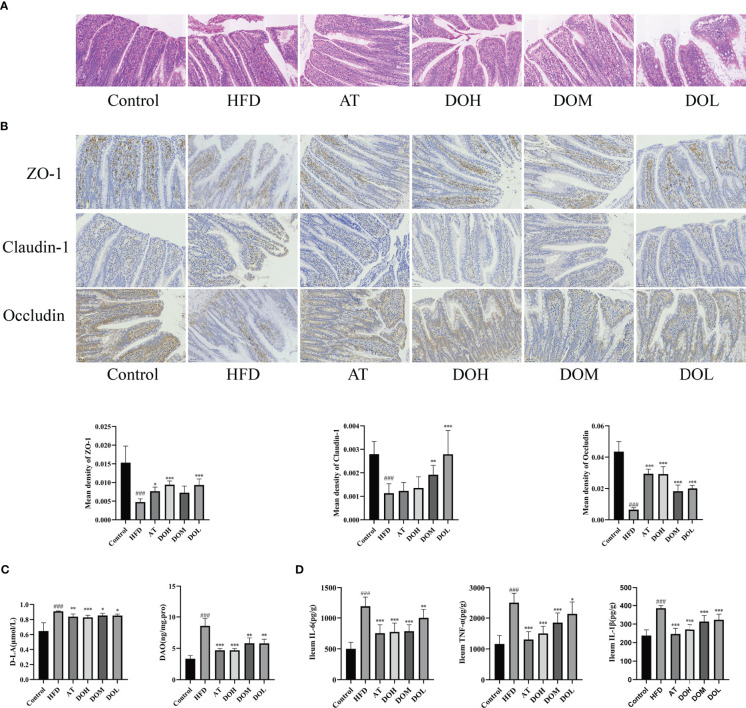
Effect of DO on intestinal permeability. **(A)** Representative images of H&E staining of the ileum by group (400x magnification); **(B)** immunohistochemistry of the ileal tight junction protein by group (400x magnification); **(C)** ileum DAO and serum D-LA levels by group; **(D)** ileum IL-6, TNF-α, IL-1β levels by group. All data are shown as the mean ± SD (n=8). ^###^ *p <* 0.001 vs. the Control group; ^*^ *p <* 0.05, ^**^ *p <* 0.01, ^***^ *p <* 0.001 vs. the HFD group.

The gut microbiota plays an important role in maintaining the integrity of the intestinal mucosal barrier, and intestinal tight junction protein is the principal determinant of intestinal permeability. Each layer of the ileum tissue in the Control and the AT groups was clearly structured, the mucosal epithelium was intact, cell morphology was normal, the intestinal villi were evenly distributed, the intestinal glands were abundant and tightly arranged, and no obvious abnormalities were found. In contrast, there was necrotic cellular debris in the intestinal lumen of the HFD group and the apical epithelium of the intestinal villi was separated from the lamina propria. This separation was much rarer in rats receiving different doses of DO (**Figure 7A**). Expression of the tight junction proteins, ZO-1, claudin-1, and occludin was significantly lower in the HFD group than in the Control group (*p*-value <0.001), and all three proteins were significantly increased following DO intervention (*p*-value<0.05, <0.01, and <0.001, respectively) (**Figure 7B**). Ileum DAO and serum D-LA levels were significantly higher in the HFD group (*p*-value <0.001), and DAO and D-LA levels were significantly lower after DO and AT treatment (*p*-value <0.05, <0.01, and <0.001, respectively) (**Figure 7C**). Ileum IL-6, TNF-α, and IL-1β levels were all significantly higher in the HFD group than in the Control group (*p*-value <0.001), and significantly decreased following DO and AT treatment (*p*-value <0.05, <0.01, and <0.001, respectively) (**Figure 7D**). The protective effect of DO on intestinal permeability was manifested by increased tight junction protein expression and lower DAO activity and D-LA and inflammatory cytokine production.

### 3.7 Effect of DO on liver inflammation

At the phylum level, Proteobacteria were most dramatically changed after 10 weeks of HFD, suggesting that there was a concomitant rise in LPS. Disruption of the gut microbiota and increased intestinal permeability allow LPS to enter the liver. TLR4 and NF-κB are important proteins related to liver inflammation during NASH, and IL-6, IL-1β, and TNF-α were the major inflammatory cytokines induced by TLR4 and NF-κB.

**Figure 8:**
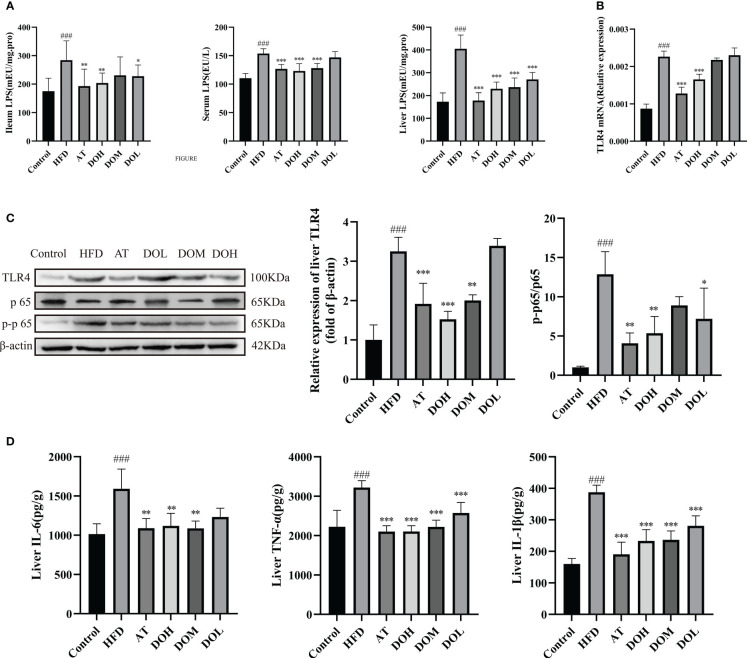
Impact of DO treatment on liver inflammation. **(A)** Ileum, serum, and liver LPS levels by group; **(B)** liver relative TLR4 mRNA expression by group; **(C)** liver TLR4 protein expression and NF-κB nuclear translocation by group; **(D)** liver IL-6, TNF-α, IL-1β levels by group. A and D results are shown as the mean ± SD (n=8), and B and C results are shown as the mean ± SD (n=3). ^###^ *p <* 0.001 vs. the Control group; *p < 0.05, **p < 0.01, ***p < 0.001 vs. the HFD group.

LPS levels in the ileum, serum and liver were significantly higher in rats in the HFD group than in the Control group (*p*-value <0.001) (**Figure 8A**), and relative TLR4 mRNA expression in the liver was significantly increased (*p*-value <0.001) (**Figure 8B**). DO and AT treatment resulted in significantly lower LPS levels in the ileum, serum, and liver (*p*-value <0.05, <0.01 and <0.001, respectively) and reduced relative TLR4 mRNA expression (*p*-value <0.001). Protein expression of TLR4 and the p-p65/p65 ratio were significantly higher in the HFD group than in the Control group (*p*-value <0.001) and both were decreased following DO and AT treatment (*p*-value <0.05, <0.01, and <0.001, respectively) (**Figure 8C**). IL-6, IL-1β, and TNF-α levels were significantly higher in the HFD group than in the Control group (*p*-value <0.001) and were significantly decreased following DO and AT treatment (*p*-value <0.01 and <0.001, respectively) (**Figure 8D**). These results suggested that DO was able to attenuate liver inflammation.

### 3.8 Correlation between gut microbiota and biochemical factors and LPS

**Figure 9:**
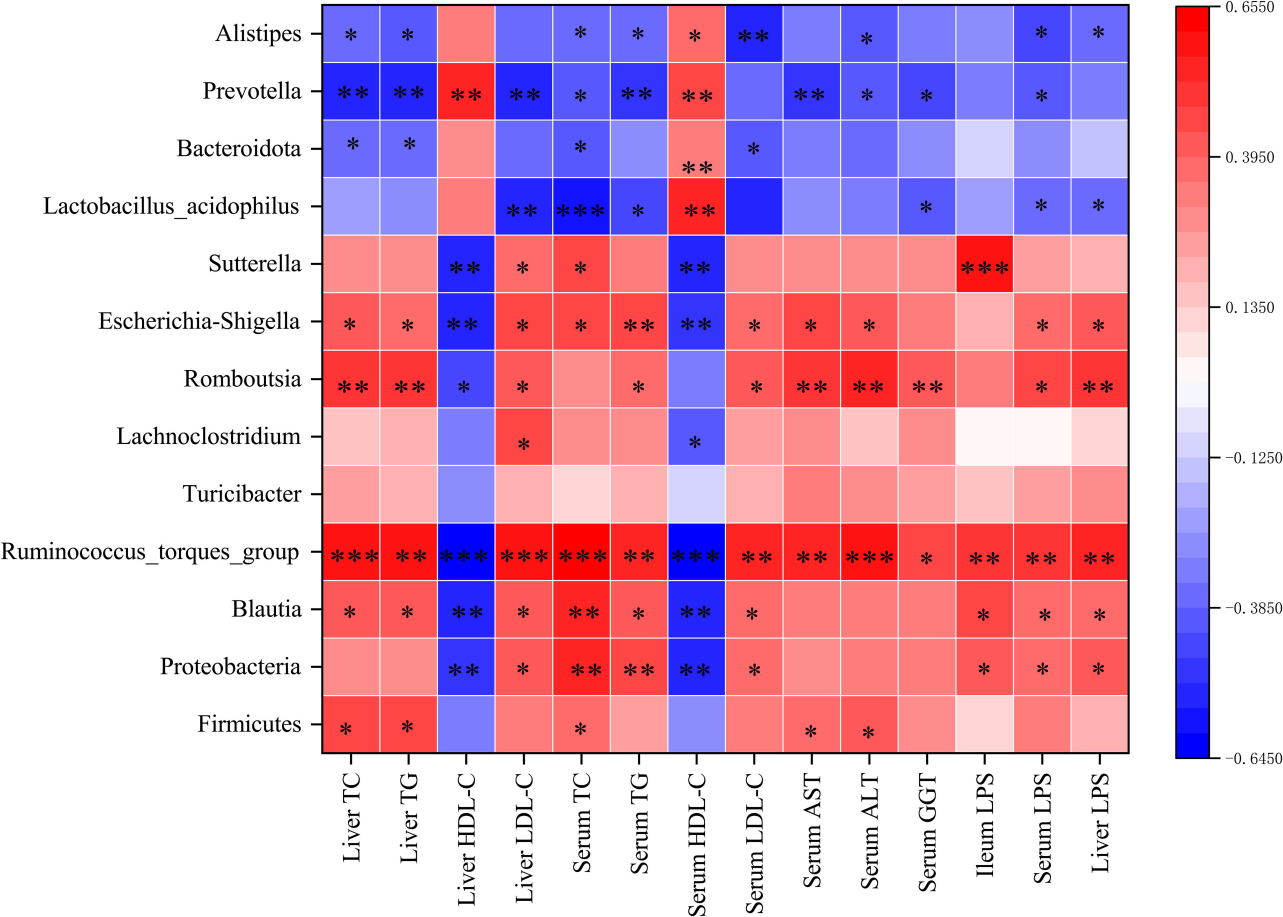
**(A)** Heatmap of the Spearman correlation between gut microbiota and the levels of biochemical factors and LPS. The colour intensities represent the degree of the associations. *p < 0.05, **p < 0.01, ***p < 0.001.

Spearman correlation analysis was performed to assess the potential correlation between gut microbiota and the levels of biochemical factors and LPS. The abundances of Firmicutes, Proteobacteria, *Romboutsia, Turicibacter, Lachnoclostridium, Blautia, Ruminococcus_torques_group, Sutterella*, and *Escherichia-Shigella* were positively correlated with TG, TC, LDL-c, AST, ALT, GGT, and LPS levels. Meanwhile, the abundances of Bacteroides, *Lactobacillus_acidophilus, Prevotella*, and *Alistipes* were positively correlated with HDL-c levels (**Figure 9A**).

## 4 Discussion

The current study established a HFD-induced NASH model in rats to investigate the effect of DO treatment on NASH and characterize the underlying mechanism caused by changes in the gut microbiota. AT, the positive control, was able to lower serum pro-inflammatory cytokine production, reduce serum cholesterol, hepatic free cholesterol, serum alpha-fetoprotein (AFP) and ALT levels, and ameliorate NASH (Domech et al., 2021; Zhang X, et al., 2021). While HFD changed the community composition of the gut microbiota and caused NASH, DO treatment regulated the gut microbiota and mitigated the disease.

NASH is associated with disordered gut microbiota, including decreased richness and diversity. Thus, improving these elements can be used to treat this disease (Boursier et al., 2016; Yan et al., 2022). To investigate the mechanism by which DO treats NASH, 16S rRNA gene sequencing was used to identify the composition of the gut microbiome in different groups of rats. DO treatment was able to prevent NASH by increasing the richness, diversity, and evenness of the gut microbiota.

At the phylum level, HFD resulted in a significant increase in the abundance of Firmicutes and Proteobacteria and a significant decrease in the abundance of Bacteroidetes. Firmicutes and Bacteroidetes are involved in energy absorption, and an unbalanced proportion of these bacteria is associated with obesity (Tenorio-Jiménez et al., 2020). Firmicutes were shown to exacerbate NAFLD severity by modulating hepatic lipid metabolism after Firmicutes were isolated from healthy individuals and inoculated into HFD-fed germ-free mice (Chen et al., 2019). NASH patients have a higher abundance of Proteobacteria, Gram-negative bacteria, including the pathogens *Escherichia-Shigella* and *Escherichia-coli*, whose outer membrane is composed of LPS (Rizzatti et al., 2017; Delik et al., 2022). In addition, Proteobacteria DNA isolated from morbidly obese patients was associated with severe liver pathology (Sookoian et al., 2020). Changes in gut microbiota promote the development of NASH. While changes in the abundance of Firmicutes and Bacteroides were not statistically significant after DO intervention, the abundance of Proteobacteria decreased significantly. These results suggest that the preventive effect of DO on NASH is associated with the regulation of Proteobacteria.

At the genus and species level, changes in microbiota richness and diversity during NASH were primarily associated with *Romboutsia* (Zeng et al., 2019), *Turicibacter*, *Lachnoclostridium* (Li et al., 2022), *Blautia* (Vallianou et al., 2021), *Ruminococcus_torques_group*, *Sutterella*, *Escherichia-Shigella, Prevotella*, *Alistipe*, and *Lactobacillus_acidophilus*. *Escherichia-Shigella* produces ethanol that can damage the intestinal mucosa and promote liver inflammation (Zhu et al., 2013), *Sutterella* has pro-inflammatory effects on the gastrointestinal tract (Hiippala et al., 2016), and *Escherichia-Shigella* and *Sutterella*, both belonging to the Proteobacteria phylum, induce LPS biosynthesis (Song et al., 2017; Xu et al., 2018).

LPS was significantly correlated with NASH in NAFLD patients and this confirmed the importance of dysbiosis during hepatic inflammation, as well as fat deposition (Hegazy et al., 2020). HFD, gut microbiota disorders, and specific physiological concentrations of LPS affect the expression and distribution of tight junctions in the intestinal mucosa and increase intestinal permeability, an early event associated with the development of NASH (Binienda et al., 2020; Chopyk and Grakoui, 2020; Rohr et al., 2020; Stephens and von der Weid, 2020). Intestinal permeability is primarily affected by tight junction proteins such as occludin, claudin-1, and ZO-1 while DAO and D-LA levels reflect the function and permeability of intestinal barriers (Mouries et al., 2019). Low intestinal permeability can prevent antigens, endotoxins, pathogens, and pro-inflammatory substances from infiltrating the body (Maciejewska et al., 2019; Zhang H, et al., 2021). LPS also specifically activates TLR4, an important inflammatory receptor in the liver, which promotes NF-κB nuclear entry and the release of inflammatory cytokines and accelerates the development of NASH (Leng et al., 2022).

**Figure 10:**
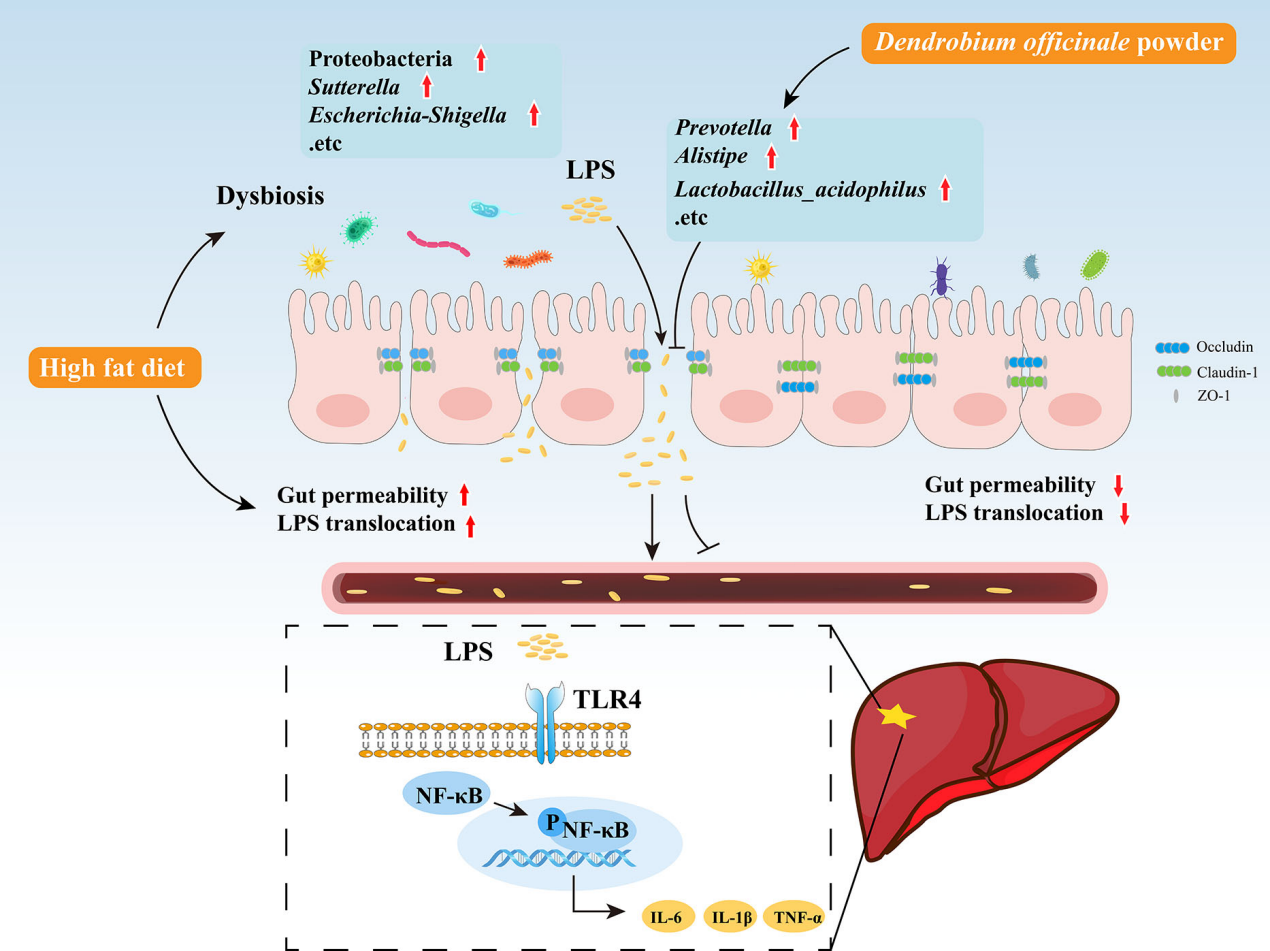
Graphic summary of the study.

The current study found that the abundance of the LPS-producing Gram-negative bacteria Proteobacteria was significantly increased in NASH rats and was accompanied by higher levels of intestinal LPS. Bacteroidetes, a Gram-negative bacteria, was negatively correlated with LPS, suggesting that gut-derived LPS is mainly produced by Proteobacteria. Intestinal epithelial cells and tight junctions were damaged while intestinal permeability and inflammation were increased in the HFD group, which may be explained by the significant enrichment of *Turicibacter*, *Ruminococcus*, *Escherichia-Shigella*, and *Sutterella*, bacteria known to disrupt the intestinal barrier (Hänninen et al., 2018; Li et al., 2020). Changes in intestinal permeability allow gut-derived LPS to enter the liver through the portal vein, increasing LPS levels in the serum and liver. Excess LPS activates liver TLR4, promoting NF-κB nuclear translocation and the release of inflammatory factors. DO treatment protects intestinal epithelial cells and tight junctions from damage, thereby reducing intestinal permeability, inhibiting liver TLR4 and NF-κB activation, and lowering inflammatory cytokine production. The reduction in liver inflammation may be related to the decreased abundance of LPS-producing bacteria, Proteobacteria, *Sutterella*, and *Escherichia-Shigella*, the intestinal barrier-disrupting bacteria, *Turicibacter*, *Ruminococcus*, and the increased abundance of *Lactobacillus_acidophilus* following DO treatment (**Figure 10**). *Lactobacillus acidophilus* is shown to regulate gut microbiota and intestinal permeability, reduce endotoxemia and inhibit TLR4/NF-κB signaling, attenuating NASH progression (Lee et al., 2021; Chen et al., 2022; Kang et al., 2022).

NASH occurs in the liver, but its pathogenesis is complex. TCM is safe, comprehensive, and effective, the holistic and multi-target function of TCM may thus be an appropriate option for NASH treatment (Zhang et al., 2020). Indeed, the ability of DO to improve NASH by regulating gut microbiota is reflective of the characteristics of TCM.

## 5 Conclusion

In summary, findings from the current study indicated that DO could regulate gut microbiota, intestinal permeability, and liver inflammation to alleviate NASH. DO treatment alleviated microbiota dysbiosis and reduced the abundance of the LPS-producing bacteria, Proteobacteria, *Sutterella*, and *Escherichia-Shigella*, reduced the abundance of the intestinal barrier-disrupting bacteria, *Turicibacter*, *Ruminococcus*, decreased intestinal permeability to reduce the movement of gut-derived LPS from the portal vein blood into the liver, inhibiting hepatic TLR4 activation and NF-κB nuclear translocation, and improving hepatic inflammation and steatosis to prevent NASH. The results also found that *Lactobacillus_acidophilus* may play a critical role during NASH. Additional follow-up, including sterility testing, is needed to further investigate the effect of DO treatment on gut microbiota with the potential mechanism of action required to prevent NASH. This study may provide theoretical support for the clinical promotion of DO.

## Data availability statement

The datasets presented in this study can be found in online repositories. The names of the repository/repositories and accession number(s) can be found below: NCBI Sequence Read Archive (SRA) database with the accession number PRJNA872008.

## Ethics statement

The animal study was reviewed and approved by The Animal Ethics Committee of the Yunnan University of Chinese Medicine.

## Author contributions

GT and SZ designed and conceptualized the study. GT analyzed the data, drafted the manuscript, carried out the statistical analysis, and interpreted the data. GT and EX carried out animal experiments. SZ, WW, and WC reviewed experimental protocols and revised the manuscript. All authors contributed to the article and approved the submitted version.
