# Supplementary material for: Dendrobium officinale alleviates high-fat diet-induced nonalcoholic steatohepatitis by modulating gut microbiota
Source: Front Cell Infect Microbiol. 2023 Feb 13;13:1078447. doi: 10.3389/fcimb.2023.1078447 (PMC9968977; doi:10.3389/fcimb.2023.1078447)
Supplement: Supplementary file 1 [file DataSheet_1.docx]

Supplementary Material

# **Supplementary method**

## **Predicting the targets of NASH**

“NASH” and “nonalcoholic fatty liver disease” were used as the keywords to obtain NASH-related targets. The targets associated with NASH were derived from GeneCards ([https://www.genecards.Org/](https://www.genecards.org/)). We acquired 785 genes after removing duplicates (Relevance score＞30).

## Screening of active compounds of *Dendrobium officinale*

We used the literature mining method (PubMed: [http://www.ncbi.nlm.nih.gov](http://www.ncbi.nlm.nih.gov/), CNKI: [http://www.cnki.net](http://www.cnki.net/)) to search active components of *Dendrobium officinale* (Chen et al., 2021; He et al., 2022; Li et al., 2022; Wei et al., 2022). The screening rules for SwissADME (http://www.swissadme.ch/) were high gastrointestinal absorption (GI absorption) properties in the pharmacokinetics column and at least two “yes” for the first five conditions in the drug-likeness column. After screening by SwissADME online tools, We obtained 48 active compounds (table 1).

## Targets for the active compounds

we explored the drug targets for the aforementioned candidate active components in *Dendrobium officinale* using SwissTargetPrediction databases (probability＞0.1) (http://www.swissadme.ch/), which resulted in a total of 623 putative targets after removing duplicates.

## Protein-protein interaction (PPI) network construction and hub gene analysis

We crossed 623 *Dendrobium officinale* active component targets with 785 NASH-related targets using VENNY 2.1 ([https://bioinfogp.cnb.csic.es/tools/venny/index.html](https://bioinfogp.cnb.csic.es/tools/venny/index.html)) to acquire the common 140 targets for *Dendrobium officinale* bioactive compound targets and the NASH associated targets (figure 1A). The online software STRING (https://cn.string-db.org/) was used to obtain PPI data, where the species was limited to “Homo sapiens,” medium confidence of protein interaction data with a score >0.400, and other basic settings were the default value (figure 1B). The PPI network was constructed with the Cytoscape 3.9.1, the higher the rank of topology analysis the darker the colour (table 2 and figure 1C). The KEGG pathway enhancement of proteins taking part in the PPI network was analyzed with the DAVID database (figure 1D).

## *Dendrobium officinale*-compounds-targets-NASH correlation network construction

The topological analysis was performed using Cytoscape 3.9.1 software, and the results showed that the network consisted of 189 nodes and 793 edges. The top ten active ingredients according to the topological analysis were Naringenin, 3′,4-Dihydroxy-3,5′-dimethoxystilbene, Dendrophenol, 4,4'-Dihydroxy-3,5-dimethoxydihydrostilbene, orchinol, Chrysoeriol, Isorhamnetin, Chrysotoxene, Erianin, Cinnamoyltyramine (table 3 and figure 2E).

## Molecular Docking

We obtained the three-dimensional structures of hub active compounds and TLR4 (PDB ID:3FXI) PDB format from the PubChem (https://pubchem.ncbi.nlm.nih.gov/) and Protein Data Bank databases (https://www.rcsb.org/). Water molecules and ligands of the target proteins were removed using PyMOL software. Next, the target proteins were hydrogenated and converted to PDBQT format. AutoDock software was used to obtain molecular docking results. Finally, the PyMOL software was used to visualize the results. Binding energy ＜–5.0 kJ/mol indicated good binding activity.

# Supplementary Tables

**Supplementary Table 1** Chemical information of 48 active compounds in *Dendrobium officinale*.

| **No** | **Compound** | **Chemical formula** | **No** | **Compounds** | **Chemical formula** |
| --- | --- | --- | --- | --- | --- |
| 1 | 3, 4-Dihydroxybenzoic acid | C7H6O4 | 25 | orchinol | C_16_H_16_O_3_ |
| 2 | Naringenin | C15H12O5 | 26 | Isovitexin | C_21_H_20_O_10_ |
| 3 | 5-Hydroxytryptophan | C11H12N2O3 | 27 | Eriodictyol | C_15_H_12_O_6_ |
| 4 | Swainsonine | C8H15NO3 | 28 | Chrysoeriol | C_16_H_12_O_6_ |
| 5 | Caffeic acid | C9H8O4 | 29 | Isorhamnetin | C_16_H_12_O_7_ |
| 6 | Serotonin | C10H12N2O | 30 | Moupinamide | C_18_H_19_NO_4_ |
| 7 | Isoacitretin | C21H26O3 | 31 | Cyanidin 3-O-rutinoside | C_27_H_31_O_15_+ |
| 8 | Vanillin | C8H8O3 | 32 | Confusarin | C_17_H_16_O_5_ |
| 9 | Caffeine | C8H10N4O2 | 33 | Nudol | C_16_H_14_O_4_ |
| 10 | Desipramine | C18H22N2 | 34 | Chrysotoxene | C_18_H_18_O_5_ |
| 11 | Triptophenolide | C20H24O3 | 35 | 3′, 4-Dihydroxy-3, 5′-dimethoxystilbene | C_16_H_16_O_4_ |
| 12 | Sanguinarine | C20H14NO4+ | 36 | Erianin | C_18_H_22_O_5_ |
| 13 | Methyl cinnamate | C10H10O2 | 37 | Dendrophenol | C_17_H_20_O_5_ |
| 14 | 4-Hydroxycinnamic Acid | C9H8O3 | 38 | 4, 4'-Dihydroxy-3, 5-dimethoxydihydrostilbene | C_16_H_18_O_4_ |
| 15 | ferulic acid | C10H10O4 | 39 | Dendrocandin A | C_17_H_20_O_5_ |
| 16 | P-Coumaroyltyramine | C17H17NO3 | 40 | Dendrocandin C | C_16_H_18_O_5_ |
| 17 | Batatasin III | C15H16O3 | 41 | Dendrocandin D | C_17_H_20_O_5_ |
| 18 | Chrysotobibenzyl | C19H24O5 | 42 | 3-O-Methylgigantol | C_17_H_20_O_4_ |
| 19 | 2-Butyl-1, 2-benzisothiazolin-3-one | C11H13NOS | 43 | Dendrobine | C_16_H_25_NO_2_ |
| 20 | Gigantol | C16H18O4 | 44 | Syringic acid | C_9_H_10_O_5_ |
| 21 | dihydroresveratrol | C14H14O3 | 45 | Vanillic acid | C_8_H_8_O_4_ |
| 22 | (S)-2-(3, 5-Dihydroxyphenyl)-5, 7-dihydroxychroman-4-one | C15H12O6 | 46 | 3-(4-Hydroxyphenyl)Propionic Acid | C_9_H_10_O_3_ |
| 23 | Cinnamoyltyramine | C17H17NO2 | 47 | 4-Hydroxybenzoic acid | C_7_H_6_O_3_ |
| 24 | 5, 7, 3', 5'-Tetrahydroxyflavanone | C15H12O6 | 48 | Ferulic acid | C_10_H_10_O_4_ |

**Supplementary Table 2** Topological analysis of hub gene.

| **No** | **Gene Symbol** | **Degree** | **Betweenness** | **Closeness** |
| --- | --- | --- | --- | --- |
| 1 | ALB | 110 | 3025.5977 | 0.8224852 |
| 2 | AKT1 | 93 | 1066.097 | 0.7433155 |
| 3 | TNF | 91 | 1051.6533 | 0.73544973 |
| 4 | VEGFA | 83 | 471.70065 | 0.69154227 |
| 5 | EGFR | 78 | 451.59598 | 0.6780488 |
| 6 | SRC | 77 | 514.7385 | 0.6682692 |
| 7 | ESR1 | 72 | 586.3779 | 0.6556604 |
| 8 | PPARG | 69 | 914.1578 | 0.6556604 |
| 9 | STAT3 | 69 | 220.55031 | 0.640553 |
| 10 | HIF1A | 66 | 300.09915 | 0.63761467 |
| 11 | PTGS2 | 65 | 494.54297 | 0.6465116 |
| 12 | MMP9 | 63 | 279.82434 | 0.6347032 |
| 13 | ERBB2 | 63 | 229.93257 | 0.6289593 |
| 14 | MTOR | 61 | 176.2443 | 0.62053573 |
| 15 | MAPK1 | 59 | 198.8248 | 0.6123348 |
| 16 | TLR4 | 54 | 190.17096 | 0.6043478 |
| 17 | MMP2 | 50 | 155.18727 | 0.5991379 |
| 18 | MAPK8 | 49 | 90.738625 | 0.8224852 |
| 19 | JAK2 | 48 | 90.90127 | 0.7433155 |
| 20 | PIK3CA | 48 | 74.28638 | 0.73544973 |

**Supplementary Table 3** Topological analysis of top 10 active compounds.

| **No** | **Compound** | **Degree** | **Betweenness** | **Closeness** |
| --- | --- | --- | --- | --- |
| 1 | Naringenin | 37 | 1080.658 | 0.42437923 |
| 2 | 3′,4-Dihydroxy-3,5′-dimethoxystilbene | 36 | 921.5217 | 0.4224719 |
| 3 | Dendrophenol | 35 | 736.1726 | 0.42058167 |
| 4 | 4,4'-Dihydroxy-3,5-dimethoxydihy drostilbene | 35 | 655.02167 | 0.42058167 |
| 5 | orchinol | 34 | 645.36505 | 0.41870824 |
| 6 | Chrysoeriol | 34 | 754.03186 | 0.41870824 |
| 7 | Isorhamnetin | 32 | 668.2499 | 0.41501105 |
| 8 | Chrysotoxene | 32 | 591.3429 | 0.41501105 |
| 9 | Erianin | 31 | 562.1304 | 0.41318682 |
| 10 | Cinnamoyltyramine | 28 | 591.5469 | 0.4078091 |

**Supplementary Table 4** Outcomes of molecular docking

| **No** | **TLR4** | |
| --- | --- | --- |
|  | **Compound** | **Docking affinity (kcal/mol)** |
| 1 | Naringenin | -5.79 |
| 2 | 3′,4-Dihydroxy-3,5′-dimethoxystilbene | -3.9 |
| 3 | Dendrophenol | -2.82 |
| 4 | 4,4'-Dihydroxy-3,5-dimethoxydihy drostilbene | -3.32 |
| 5 | orchinol | -6.21 |
| 6 | Chrysoeriol | -3.81 |
| 7 | Isorhamnetin | -3.38 |
| 8 | Chrysotoxene | -6.2 |
| 9 | Erianin | -2.9 |
| 10 | Cinnamoyltyramine | -7.47 |

# Supplementary Figures


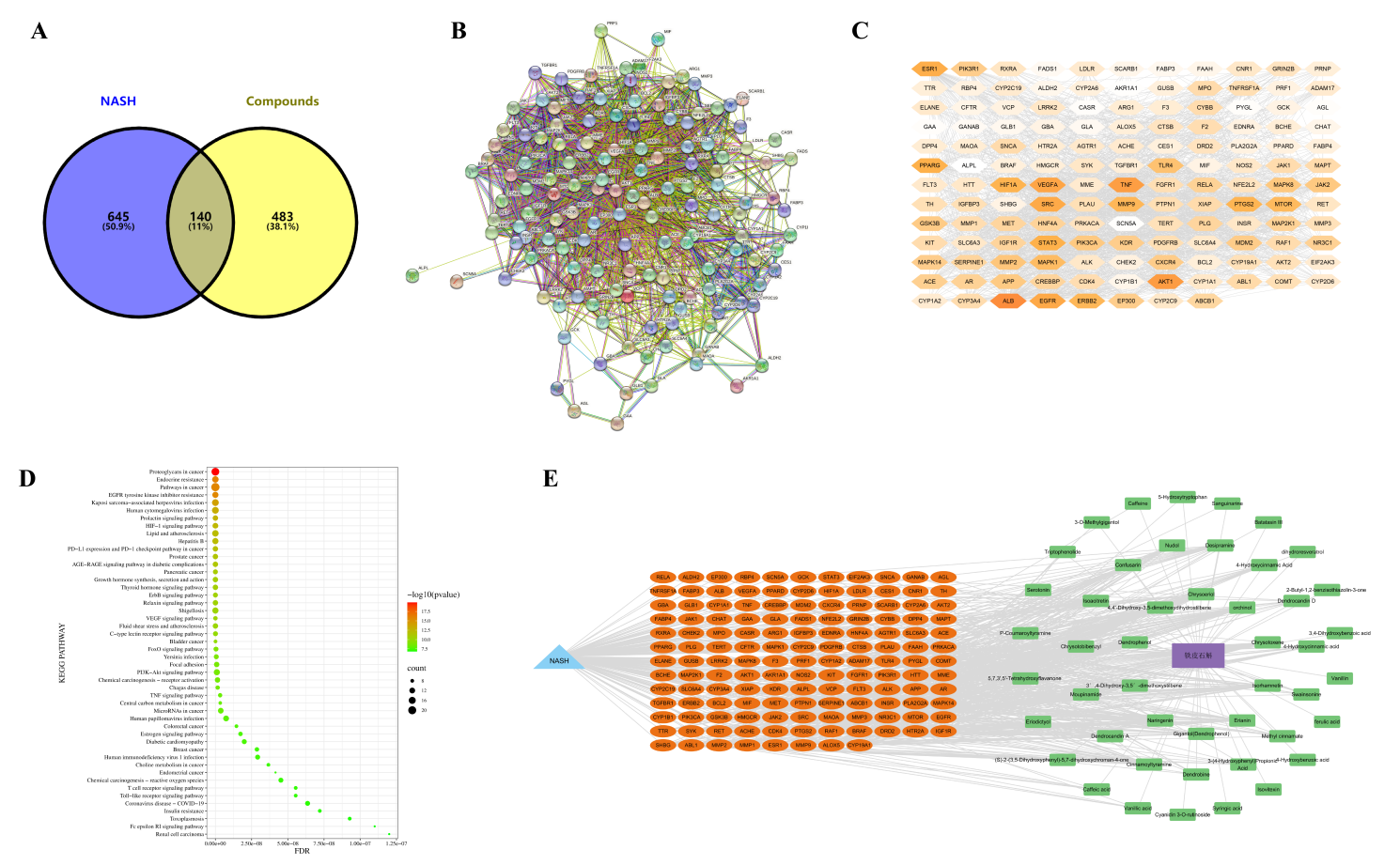


**Supplementary Figure 1.** The Mechanism of *Dendrobium officinale* as a Treatment for NASH based on network pharmacology. (A)Overlapping targets of active component targets and NASH targets. (B)Protein-protein interaction (PPI) network analysis. (C)The PPI network was constructed with Cytoscape 3.9.1, the higher the rank, the darker the colour. (D)KEGG enrichment analysis was performed on overlapping targets. (D)*Dendrobium officinale*-compounds-targets-NASH correlation network. The Blue triangle is NASH, the orange circle is the potential target, the purple square is *Dendrobium officinale*, and the green square is the active ingredient. The connection line between the nodes represents the corresponding relationship between the two.


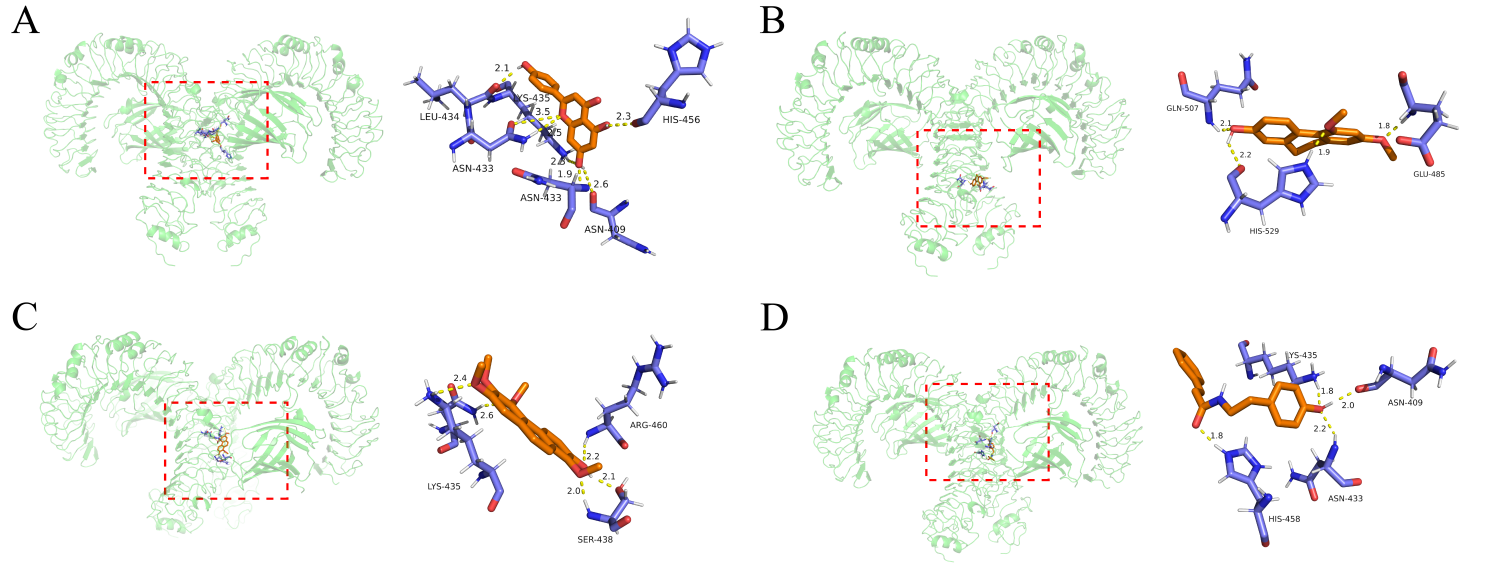


**Supplementary Figure 2.** Molecular docking diagrams of TLR4 with Naringenin (A), orchinol(B), Chrysotoxene(C), and Cinnamoyltyramine (D).

**Reference**

CHEN, W., LU, J., ZHANG, J., WU, J., YU, L., QIN, L., ZHU, B. (2021). Traditional Uses, Phytochemistry, Pharmacology, and Quality Control of *Dendrobium officinale* Kimura et. Migo. *Front. Pharmacol*. 12: 726528. doi: 10.3389/fphar.2021.726528

HE, Y., LI, L., CHANG, H., CAI, B., GAO, H., CHEN, G., et al. (2022). Research progress on extraction, purification, structure and biological activity of *Dendrobium officinale* polysaccharides. *Front. Nutr*. 9:965073. doi: 10.3389/fnut.2022. 965073

LI, L. Z., WANG, H. Y., HUANG, J. H., LIU, K., FENG, X. J., WANG, X. M., et al. (2022). The Mechanism of *Dendrobium officinale* as a Treatment for Hyperlipidemia Based on Network Pharmacology and Experimental Validation. *Evid. Based. Complement. Alternat. Med**.* 2022: 5821829. doi:10.1155/2022/5821829

WEI, L., DONG, W., HAN, Z., CHEN, C., JIN, Q., HE, J., et al. (2022). Network Pharmacologic Analysis of *Dendrobium officinale* Extract Inhibiting the Proliferation of Gastric Cancer Cells. *Front. Pharmacol.* 13: 832134. doi:10.3389/fphar. 2022. 832134. doi:10.3389/fphar.2022.832134
